# The Phenomenology of the Chromic Response in Transition-Metal Oxides

**DOI:** 10.3390/ma19122610

**Published:** 2026-06-17

**Authors:** Alexandru Varzari, Gheorghe Ghilețchii, Ştefan-Andrei Irimiciuc, Ján Lančok, Sergiu Vatavu

**Affiliations:** 1 Physics of Semiconductors and Devices Lab, Faculty of Physics and Engineering, Moldova State University, 2009 Chisinau, Moldova; alexandru.varzari@usm.md (A.V.); ghiletchii.gheorghe@usm.md (G.G.); sergiu.vatavu@usm.md (S.V.); 2 Institute of Physics of the Czech Academy of Sciences, 182 00 Prague, Czech Republic; irimiciuc@fzu.cz; 3 National Institute for Laser, Plasma and Radiation Physics, 077125 Magurele, Romania

**Keywords:** chromism, thermochromism, electrochromism, transition-metal oxides, VO_2_, metal–insulator transition, small polarons, Drude–Lorentz model

## Abstract

Chromic materials exhibiting reversible changes in optical properties under external stimuli represent an important class of smart materials with applications in smart windows, sensors, and optoelectronic devices. Transition-metal oxides (TMOs) provide a versatile platform for chromic functionality due to their coupled structural, electronic, and optical properties. In this review, the chromic response of selected TMO thin films is analyzed using both microscopic and phenomenological approaches. The microscopic description is based on many-body theory, including Green’s function methods and correlation effects, while the macroscopic optical response is described using Drude–Lorentz and Tauc–Lorentz models within the effective medium approximation. Chromic behavior in TMOs is shown to originate from two principal mechanisms: (i) electronic and structural reconstruction driven by Peierls–Mott metal–insulator phase transitions, leading to thermochromism (notably in VO_2_ and V_2_O_3_), and (ii) formation of localized states driven by small-polaron injection, giving rise to electrochromism, gasochromism, and photochromism. The models are applied to representative systems, including VO_2_, WO_3_, NiO, and TiO_2_, demonstrating the chromic changes in the dielectric function spectra. These results highlight chromism in TMOs as a multiscale phenomenon linking microscopic interactions with macroscopic optical response.

## 1. Introduction

The phenomenon of chromism refers to a reversible modification of the optical response of a material under the action of an external stimulus. It corresponds to a stimulus-induced variation in absorption, reflection, and transmission. Depending on the nature of the external stimulus, the most common types of chromism include, among others, thermochromism, electrochromism, gasochromism, and photochromism, driven by temperature, electric field or voltage, gas concentration, and light radiation, respectively.

Chromic materials represent an important class of stimulus-responsive smart materials whose optical properties can be actively modulated [1,2,3]. This functionality makes them highly relevant for applications in smart windows [4], sensors [5], camouflage [6], and optoelectronic devices [7]. In practical applications, the performance of chromic materials is determined not only by the magnitude of optical modulation, but also by reversibility, response time, cycling stability, durability and spectral selectivity.

Among different classes of chromic materials, transition-metal oxides (TMOs) occupy a special position because their optical response is strongly coupled to their electronic, structural, defect-related, and oxygen stoichiometry [8,9,10]. These compounds typically consist of transition-metal cations (e.g., V^4+^, W^6+^, Ni^2+^) and oxygen anions (O^2−^), where the electronic structure is governed by the interplay between transition-metal *d* states and oxygen 2p orbitals. Stoichiometric TMOs are often wide-bandgap dielectrics; however, depending on composition and electronic structure, they can exhibit either high transparency or strong absorption in the visible and near-infrared spectral range [11]. Strong electron–phonon interactions and narrow *d* bands promote local lattice distortions, typically within MO_6_ octahedra, and play a key role in determining their physical properties.

A broad variety of TMOs exhibit chromic functionality. In the present review, VO_2_, WO_3_, NiO, and TiO_2_ are selected as representative benchmark systems, since they cover the principal phenomenological scenarios of chromism in TMOs, including phase-transition-driven thermochromism, ion-intercalation-driven electrochromism and gasochromism, and photoinduced polaronic optical response. VO_2_ represents a reference thermochromic oxide in which the optical response is governed by a temperature-driven metal–insulator transition [12,13,14]. Tungsten oxide WO_3_ is one of the most widely studied cathodic electrochromic and gasochromic oxides, where coloration is associated with ion intercalation and the formation of W^5+^/W^6+^ polaronic states [15,16]. Nickel oxide NiO is a typical anodic electrochromic material, with optical modulation related to hole polaron formation and changes in the Ni^2+^/Ni^3+^ ratio [15,17]. Titanium dioxide TiO_2_, especially in amorphous, nanocrystalline, or defect-rich forms, is relevant for photochromic and defect-mediated optical responses involving Ti^3+^ centers and oxygen vacancies [15,18].

Other oxides further expand this phenomenology. V_2_O_3_ exhibits a strong MIT with pronounced optical contrast, primarily associated with electronic correlations, although its relatively low transition temperature limits direct room-temperature chromic applications [19,20,21]. V_2_O_5_, together with WO_3_, is widely considered for gas-responsive and hydrogen-sensing applications, where optical modulation is related to redox processes, ion insertion, and polaronic states [15,22,23,24,25]. MoO_3_ and MoO_3_-based materials have recently been reviewed mainly from the perspective of gas sensors, lithium-ion batteries, adsorption, and photocatalysis; however, their layered structure, oxygen vacancy chemistry, intercalation ability, and Mo^6+^/Mo^5+^ redox states also make them relevant to electrochromic, photochromic, and gas-responsive optical phenomena [26]. Nb_2_O_5_ has been reviewed primarily in the context of polymorphism and electrical energy storage applications, where different crystal structures control ion migration and electrochemical performance; in the context of chromic oxides, it may therefore be regarded as an emerging ion-intercalation and defect-sensitive system [27].

VO_2_ is mainly associated with thermochromic smart windows and optical switching, because its temperature-driven MIT provides strong modulation of NIR transmittance while preserving partial transparency in the VIS range [28,29,30]. For practical applications, the key challenge is to optimize the transition temperature, visible luminous transmittance, solar modulation ability, hysteresis width, and durability. Elemental doping is one of the most widely used strategies for tailoring the MIT temperature and optical properties of VO_2_. Depending on the dopant, this tuning may be related to changes in carrier concentration, lattice parameters, V–V dimerization, strain state, and electronic band structure, thereby affecting either the electronic or structural component of the phase transition [31,32,33,34]. Another important approach is the use of multilayer and antireflection coatings, which reduce optical reflection losses, improve luminous transmittance, and enhance overall thermochromic performance [35,36,37]. In addition to thermochromism, hydrogen incorporation can modify the valence state, local atomic structure, electron correlations, and transmittance of VO_2_, enabling gasochromic coloration and suggesting possible use in hydrogen-sensitive optical devices [38,39].

WO_3_ and NiO represent the classical complementary pair of cathodic and anodic electrochromic TMOs used in electrochromic smart windows. WO_3_ colors upon charge and ion insertion, whereas NiO exhibits anodic coloration associated with oxidation processes. In multilayer electrochromic devices, the combination of WO_3_ and NiO allows simultaneous modulation of optical transmittance and charge compensation, making this pair one of the most established oxide platforms for energy-efficient glazing and low-power optical modulation [15,40,41]. The performance of such devices is governed not only by the intrinsic electrochromic response of each oxide, but also by electrolyte selection, ion-storage layers, dopant chemistry, nanostructuring, charge balance between electrodes, interfacial stability, switching speed, and cycling durability [42,43].

These characteristics make TMOs particularly suitable for chromic functionality, as their optical response is strongly coupled to their electronic and structural state [9]. Despite extensive studies of individual chromic TMOs, the underlying mechanisms of chromism and their connection to both microscopic theory and phenomenological optical modeling remain fragmented across the literature. Therefore, the aim of this work is to provide a unified theoretical framework for chromic phenomena in TMOs, combining microscopic descriptions with phenomenological approaches to the optical response.

The optical response of chromic materials to external stimuli is governed by the complex dielectric function. From a microscopic perspective, the mechanisms responsible for modifications of the dielectric function can be broadly classified into two fundamental categories. The first involves phase transitions associated with a reconstruction of the electronic structure, such as metal–insulator transitions (MITs) [44,45]. The second mechanism is related to the injection of polaronic states, which induces local modifications of the electronic system [46,47]. From a phenomenological perspective, the dielectric function is commonly described using Drude–Lorentz or Tauc–Lorentz models [48] and fitted to experimental optical data.

In order to connect chromic functionality with optical phenomenology, it is useful to briefly outline the microscopic mechanisms that modify the electronic structure and, consequently, the complex dielectric function.

Fundamental reconstruction of the electronic system driven by structural and correlation-induced phase transitions leads to discontinuous or strongly nonlinear changes in the complex dielectric function, resulting in pronounced optical contrast. Such behavior is commonly associated with MITs [44], which can be driven by both electron–electron and electron–phonon interactions.

The Mott–Hubbard MIT is driven by electron–electron interactions and occurs when the Coulomb repulsion becomes comparable to the kinetic energy of electrons. This situation is typical for transition metal compounds due to the reduced overlap of *d* orbitals in the crystal lattice. Mott [49,50] was the first to interpret the MIT in terms of electron–electron interactions. A quantum-mechanical description of the Mott transition was later developed within the Hubbard model [51] and in related approaches proposed by Kemeny [52] and Gutzwiller [53]. Brinkman and Rice [54] described the divergence of the quasiparticle effective mass in the strong-correlation limit, thereby connecting the Mott transition with the disappearance of the quasiparticle peak. The introduction of the infinite-dimensional limit by Metzner and Vollhardt [55] laid the foundation for dynamical mean-field theory (DMFT), in which the Hubbard model is mapped onto an effective single-site problem embedded in a self-consistent medium [56,57].

The Peierls MIT is driven by electron–phonon interactions and arises from a lattice instability, typically in low-dimensional systems. In transition-metal compounds such as VO_2_, the reduced overlap of *d* orbitals leads to quasi-one-dimensional electronic structures, enabling the Peierls transition. The concept of such structural–electronic instability was first formulated by Peierls [58]. Peierls theory was further refined by Kohn [59], who demonstrated the divergence of the electronic susceptibility at $q=2k_{F}$, and by Fröhlich [60], who introduced the concept of collective electron–phonon motion. This transition is closely related to the formation of charge density waves (CDWs) [61,62].

Modern theoretical approaches extend these frameworks by combining DMFT with many-body perturbation theory (e.g., GW+DMFT) [63] and by incorporating electron–phonon coupling within density functional theory (DFT+EPC) [64]. In real TMOs, and especially in VO_2_, both electron–electron and electron–phonon interactions are often simultaneously significant. As a result, the MIT in these materials is commonly described within a combined Peierls–Mott framework. A unified description can be formulated within the Hubbard–Holstein model [65,66]. Phenomenologically, the MIT is reflected in the emergence or suppression of the Drude peak, accompanied by a redistribution of the spectral weight.

Local modification of the electronic system driven by polaron injection leads to the appearance of additional absorption channels, mainly in the NIR region, and consequently to optical contrast. In TMOs, polarons can be injected during intercalation–deintercalation processes or generated by photoexcitation. When the electron–phonon coupling energy exceeds the electronic bandwidth, an injected charge carrier, either an electron or a hole, loses its ability to delocalize and becomes self-trapped at a lattice site. In this process, the charge carrier polarizes its local environment, induces a lattice distortion, and forms a self-consistent state involving both electronic and phononic subsystems. Such a coupled carrier–lattice excitation constitutes a quasiparticle known as a polaron [46,67].

The possibility that lattice distortions can trap electrons was first proposed by Landau [68], while the first formal description of the polaron was developed by Pekar [69]. Landau and Pekar later showed that the effective mass of a polaron is enhanced [70]. Depending on the relative magnitudes of the electron–phonon coupling and the electronic bandwidth, polarons are commonly classified as large or small. Large polarons originate from long-range electron–phonon coupling and are described by Fröhlich theory [71,72]. Small polarons correspond to the short-range strong-coupling regime and are commonly described by the Holstein Hamiltonian [65,73]. In most TMOs, narrow *d* bands and strong carrier localization favor the formation of small polarons. Phenomenologically, polaron injection leads to the emergence of additional Lorentz oscillators associated with localized states.

The interplay between these microscopic mechanisms and their phenomenological optical signatures forms the basis for a unified description of chromism in TMOs. The following sections first discuss the underlying microscopic mechanisms and their manifestation in representative TMO systems, and subsequently present the phenomenological modeling of the dielectric response.

## 2. Microscopic Mechanisms of Chromism in TMOs

### 2.1. Peierls–Mott Transition

In TMOs such as VO_2_, the reduced overlap of *d* orbitals leads to the formation of quasi-one-dimensional chains, enabling the interplay of both Peierls and Mott–Hubbard mechanisms. A minimal model capturing the Peierls–Mott MIT is provided by the Hubbard–Holstein Hamiltonian [51,65,74]:

(1) $$H=-t\sum_{\langle i,j\rangle ,\sigma }\left(c_{i\sigma }^{†}c_{j\sigma }+h.c.\right)+U\sum_{i}n_{i↑}n_{i↓}+\hbar \omega_{0}\sum_{i}b_{i}^{†}b_{i}+g\sum_{i,\sigma }n_{i\sigma }\left(b_{i}^{†}+b_{i}\right)$$

The first term in Equation (1) represents the kinetic energy associated with electron hopping between lattice sites *i* and *j*. Here, $c_{i\sigma }^{†}$ and $c_{j\sigma }$ are the electron creation and annihilation operators with spin σ at sites *i* and *j*, respectively, while h.c. denotes the Hermitian conjugate term corresponding to the reverse hopping process. The hopping integral $t=\langle \psi_{i}|H|\psi_{j}\rangle$ is the matrix element of the Hamiltonian between neighboring atomic orbitals localized at sites *i* and *j*. Due to the weak overlap of d orbitals, the hopping integral typically takes small values. As a result, electrons on neighboring sites form a narrow energy band of width W≈2z|t|, where *z* is the coordination number. The bandwidth *W* characterizes the degree of electron delocalization and, consequently, the tendency toward metallic behavior.

The second term in Equation (1) describes the on-site interaction between two electrons with opposite spins (↑ and ↓) at the same lattice site *i*. Here, $n_{i\sigma }=c_{i\sigma }^{†}c_{i\sigma }$ is the electron number operator, and the parameter *U* represents the on-site Coulomb repulsion, i.e., the energetic cost of double occupancy. In systems with narrow orbitals, such as d orbitals in transition metal compounds, the Coulomb interaction becomes significant. The energy scale *U* thus characterizes the degree of electron localization and the tendency toward Mott insulating behavior.

The third and fourth terms in Equation (1) describe the phonon contribution and the electron–phonon coupling. Here, $\hbar \omega_{0}$ is the phonon frequency, $b_{i}^{†}$ and $b_{i}$ are the phonon creation and annihilation operators at site *i*, and *g* is the coupling constant that determines the strength of the interaction between the electronic density and local lattice distortions.

The momentum-averaged density of states (DOS) is determined by the single-particle spectral function [56], which describes the spectral weight of electronic excitations with quasimomentum $\hbar \vec{k}$ and energy ℏω. The spectral function is directly related to the imaginary part of the retarded Green’s function, yielding:

(2) ρ(ω)=1N∑k→A(k→,ω)=−1πN∑k→ImGR(k→,ω);GR(k→,ω)=1ω+μ−εk→−Σ(k→,ω)

Here, *N* is the number of lattice sites (or equivalently the number of $\vec{k}$-points in the Brillouin zone), $\varepsilon_{\vec{k}}$ is the electronic dispersion, μ is the chemical potential, and $\Sigma (\vec{k},\omega )$ is the electronic self-energy.

The nature of the self-energy provides a fundamental distinction between Mott–Hubbard and Peierls mechanisms of the MIT. In the Mott–Hubbard scenario, the transition is governed by local electronic correlations, and the momentum dependence of the self-energy can be neglected within dynamical mean-field theory (DMFT), i.e., $\Sigma \left(\vec{k},\omega \right)\approx \Sigma \left(\omega \right)$. In contrast, the Peierls transition is associated with a lattice-driven instability that breaks translational symmetry and leads to a momentum-dependent reconstruction of the electronic spectrum.

The real part of the self-energy, ReΣ(k→,ω), leads to a renormalization of the quasiparticle dispersion and effective mass, while its imaginary part, ImΣ(k→,ω), determines the quasiparticle lifetime and spectral broadening. In the case of the Mott–Hubbard transition, where electronic correlations are predominantly local, the momentum dependence of the self-energy can be neglected, $\Sigma \left(\vec{k},\omega \right)\approx \Sigma \left(\omega \right)$. In this regime, the effective mass renormalization is given by:

(3) m*m=Z−1=1−∂ReΣ(ω)∂ωω=0

Here, *Z* is the quasiparticle weight. The vanishing of *Z* signals the breakdown of the Fermi-liquid picture and the onset of the Mott insulating state.

Within the Hubbard–Holstein framework, the MIT is governed by the competition between electronic correlations and electron–phonon coupling. Electronic correlations are characterized by the ratio U/W, where *U* is the on-site Coulomb interaction and *W* is the electronic bandwidth. The electron–phonon interaction is described by the dimensionless coupling constant $\lambda =g^{2}/KW$, where *g* is the coupling strength and *K* is the lattice stiffness. Depending on the relative magnitudes of U/W and λ, the system may exhibit Mott–Hubbard, Peierls, or combined Peierls–Mott transitions.

**Figure 1:**
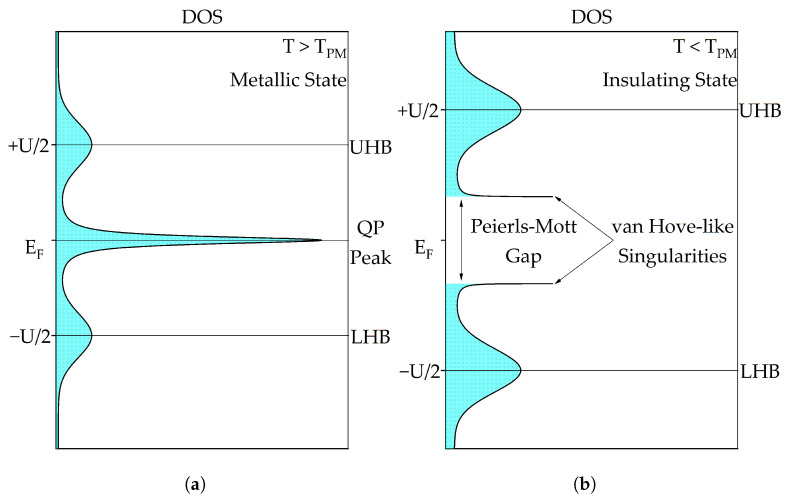
Schematic DOS for (**a**) strongly correlated metallic state, (**b**) Peierls–Mott insulating state.

The Peierls–Mott transition can be interpreted as the opening of a gap in a strongly correlated metallic state. In this scenario, the quasiparticle peak characteristic of a correlated metal (Figure 1a) is suppressed by a lattice-driven instability, resulting in a gapped density of states with reconstructed Hubbard bands (Figure 1b).

The evolution of the DOS and the self-energy $\Sigma (\vec{k},\omega )$ for a half-filled system (e.g., d^1^, d^3^) and one-dimensional tight-binding system can be classified into several regimes:

(i) Weakly correlated metal (λ≪1, U/W≪1). Electron delocalization dominates, and the self-energy is weakly frequency-dependent. The quasiparticle weight approaches unity (Z≈1, $m^{*}\approx m$), and the DOS remains finite at the Fermi level. The system behaves as a conventional Fermi liquid.

(ii) Strongly correlated metal (λ≪1, U/W∼1). Electronic correlations become significant, leading to a characteristic three-peak DOS structure [56]: a narrow quasiparticle peak at the Fermi level ($E_{F}$), and lower and upper Hubbard bands (LHB and UHB), corresponding to electron removal and addition processes, respectively. The quasiparticle peak represents the coherent part of the spectrum, while the Hubbard bands are incoherent.

(iii) Mott insulator (λ≪1, U/W≫1). Electron localization dominates, and the quasiparticle weight vanishes (Z→0, $m^{*}\to \infty$), corresponding to the Brinkman–Rice scenario [54]. The quasiparticle peak disappears, and a correlation gap of order Δ∼U−W opens between the Hubbard bands. The DOS vanishes at the Fermi level, indicating insulating behavior.

(iv) Peierls insulator (λ≳1, U/W∼1). The system becomes unstable with respect to a lattice distortion with wavevector $Q=2k_{F}$, leading to a doubling of the unit cell and opening of a gap at the Fermi level. This distortion mixes electronic states with momenta $\vec{k}$ and $\vec{k}+\vec{Q}$ and results in a transition to an insulating state.

(v) Peierls–Mott insulator (λ≳1, U/W≫1). Both electronic correlations and lattice distortions are significant. Correlations enhance electron–phonon coupling (often referred to as correlation-enhanced Peierls instability), leading to strong localization. The electronic spectrum exhibits Hubbard bands further split by lattice dimerization into bonding and antibonding subbands. The resulting gap has a mixed correlation-driven and lattice-driven origin, as observed in systems such as VO_2_.

### 2.2. Small Polaron Injection

Small-polaron formation in TMOs can occur via several mechanisms. One of the primary routes is ion intercalation–deintercalation driven by interaction with the external environment, such as electrolytes or reactive gases. Depending on the material, TMOs are commonly classified into cathodic coloration types (e.g., WO_3_) and anodic coloration types (e.g., NiO). In cathodic materials, coloration is typically associated with the intercalation of small cations (H^+^, Li^+^, Na^+^) into interstitial sites or diffusion channels of the crystal lattice, whereas anodic materials undergo the reverse process of deintercalation. These processes can be described as solid-state redox reactions [75], in which the TMOs lattice acts as a host capable of accepting or donating charge. The concept of such solid-state electrochemical processes was first introduced by Deb [76] and further developed by Faughnan [77].

Another important mechanism is carrier generation via optical excitation. An incident electromagnetic wave creates nonequilibrium charge carriers according to $h\nu \to e^{-}$ (CB) $+\;h^{+}$ (VB). Following rapid energy relaxation, the charge carrier may become self-trapped due to strong electron–phonon coupling, leading to the formation of a photoinduced small polaron. A necessary condition for this process is that the electron–phonon relaxation time is shorter than the carrier recombination time, $\tau_{e\text{-}ph}\ll \tau_{\mathrm{rec}}$. Under this condition, the excited carrier loses its excess energy and becomes localized, forming a small polaron.

**Figure 2:**
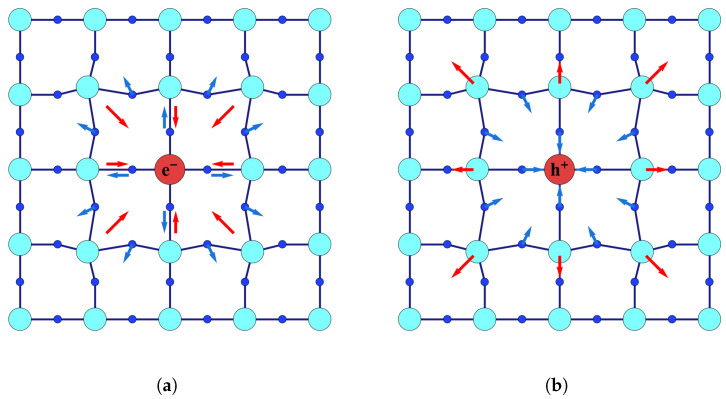
Schematic illustration of the formation of a small polaron in the case of the self-trapping of (**a**) electron and (**b**) hole. The cyan circles represent lattice ions, the red circle denotes the localized charge carrier, and the colored arrows indicate local lattice displacements around the polaron.

Injected electrons or holes in a TMO structure become localized at cation sites. Localized electrons lead to the formation of lower-valence centers (Me^*n*+^ → Me^(*n*−1)+^), whereas localized holes result in higher-valence centers (Me^*n*+^ → Me^(*n*+1)+^). As a consequence, the local electronic structure is reconstructed. These processes are accompanied by polarization of the surrounding O^2−^ anions and local lattice distortions. Such a coupled charge–lattice configuration constitutes a small polaron [78] (Figure 2).

When the condition $E_{e\text{-}ph}≳W$ is satisfied, an injected charge carrier (electron or hole) loses its ability to delocalize and becomes self-trapped at a lattice site. This indicates that the energy gain associated with local lattice distortion exceeds the kinetic energy of electronic hopping. In this process, the charge carrier polarizes its local environment, induces a lattice distortion, and forms a self-consistent state involving both electronic and phononic subsystems.

The minimal model describing small-polaron formation is given by the Holstein Hamiltonian [65,73]:

(4) $$H=-t\sum_{\langle i,j\rangle ,\sigma }\left(c_{i\sigma }^{†}c_{j\sigma }+h.c.\right)+\hbar \omega_{0}\sum_{i}b_{i}^{†}b_{i}+g\sum_{i,\sigma }n_{i\sigma }\left(b_{i}^{†}+b_{i}\right)$$

Here, the first term describes electron hopping between lattice sites, the second term corresponds to local lattice vibrations (phonons), and the third term represents the local electron–phonon interaction.

The binding energy of a small polaron, i.e., the energy gain associated with its formation, is given by $E_{p}=g^{2}/\hbar \omega_{0}$, where $E_{p}$ characterizes the stabilization of the localized state due to electron–phonon coupling. Small polarons move through the crystal lattice via a hopping mechanism. When $E_{p}\gg t$, the charge carrier becomes localized, and charge transport is no longer coherent but proceeds via thermally activated hopping, with a probability proportional to $\exp(-E_{a}/k_{B}T)$, where $E_{a}$ is the activation energy.

In the polaronic regime, the single-particle Green’s function is strongly renormalized by the electron–phonon self-energy, $\Sigma_{\mathrm{pol}}\left(\omega \right)$. This self-energy exhibits a pronounced frequency dependence and a substantial imaginary part, leading to spectral broadening and the emergence of in-gap states. As a result, the overall band structure and the global density of states remain largely preserved, while additional spectral features, such as polaronic peaks and exponential tails, appear within the bandgap. This local modification of the electronic structure distinguishes polaronic mechanisms from phase-transition-driven processes, which involve a global reconstruction of the density of states.

**Figure 3:**
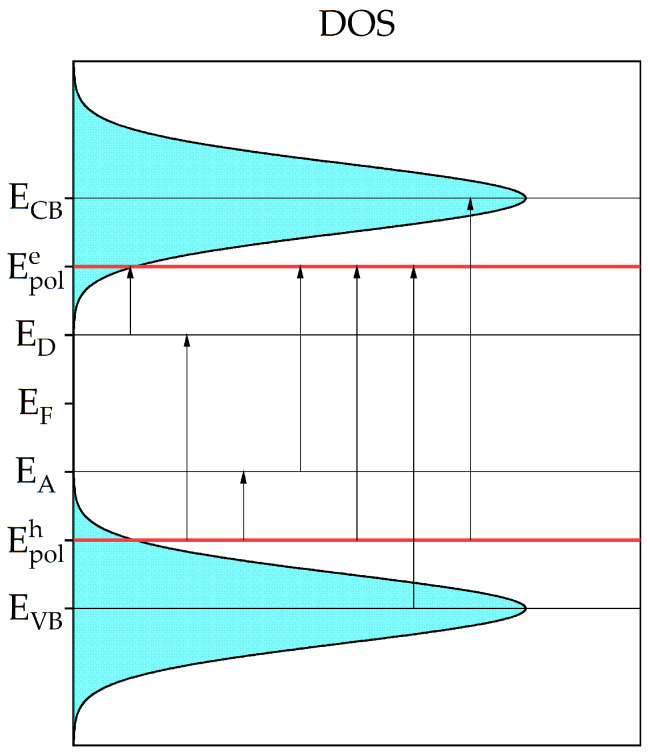
Schematic DOS with polaronic levels.

Polarons introduce localized energy levels within the bandgap of TMOs, thereby opening additional optical absorption channels. These include transitions between the valence or conduction bands and polaronic states, transitions between intrinsic defect states and polaronic levels, as well as inter-polaron transitions (small-polaron absorption) (Figure 3). Small-polaron absorption corresponds to photon-assisted hopping, i.e., intervalence charge transfer of an electron between two neighboring localized centers. This mechanism gives rise to a broad absorption band in the optical spectrum.

### 2.3. Dielectric Function and Chromism Mechanisms

The modification of the optical properties can be described in terms of the complex dielectric function:

(5) $$\varepsilon \left(\omega \right)=\varepsilon_{\infty }+\frac{i}{\varepsilon_{0}\omega }\sigma \left(\omega \right)$$

Here, $\varepsilon_{\infty }$ is the high-frequency dielectric constant and σ(ω) is the optical conductivity. Within linear response theory, the optical conductivity can be obtained from the Kubo formula in terms of the current–current correlation function.

In general, the optical conductivity can be decomposed into singular and regular contributions:

(6) $$\sigma \left(\omega \right)=D\;\delta \left(\omega \right)+\sigma^{\mathrm{reg}}\left(\omega \right)$$

Here, *D* is the Drude weight, which characterizes the coherent response of delocalized charge carriers, while $\sigma^{\mathrm{reg}}\left(\omega \right)$ corresponds to incoherent processes.

From a microscopic perspective, different chromic mechanisms manifest themselves through distinct modifications of σ(ω) and, consequently, ε(ω). In phase-transition-driven chromism, the Drude contribution emerges or vanishes, accompanied by a redistribution of spectral weight. In contrast, polaronic mechanisms lead to the suppression of the coherent Drude response and the appearance of additional absorption features associated with localized states. In the Peierls–Mott mechanism, the main control parameter is temperature. Across the critical temperature $T_{c}$, the system undergoes a transition between insulating and metallic states, which leads to a pronounced change in the dielectric function. Therefore, the corresponding chromic response is classified as thermochromism. In contrast, in the small-polaron mechanism, the dielectric function changes with the concentration of polaronic states. This concentration can be controlled by an external electric field, gas atmosphere, or optical excitation, giving rise to electrochromism, gasochromism, and photochromism, respectively. The correspondence between microscopic mechanism, control parameter, optical signature, and representative TMO systems is summarized in Table 1.

## 3. Phenomenological Modeling of the Optical Response

### 3.1. Dielectric Function and Optical Properties

The phenomenon of chromism refers to a reversible process of changing the color or transparency of a material under the influence of external factors such as temperature, an electric field, light, and other external stimuli. The physical principle underlying this phenomenon is based on a change in the optical properties of the material due to modifications in its electronic structure. The optical response of the electronic system to an external stimuli is described by the complex dielectric function $\tilde{\varepsilon }\left(\omega \right)$ [48], which is related to the complex refractive index $\tilde{n}\left(\omega \right)$ (for non-magnetic media $\tilde{\mu }\approx 1$) by the following relation [79]:

(7) $$\tilde{\varepsilon }\left(\omega \right)={\tilde{n}}^{2}\left(\omega \right);\;\tilde{\varepsilon }\left(\omega \right)=\varepsilon_{1}\left(\omega \right)+i\varepsilon_{2}\left(\omega \right);\;\tilde{n}\left(\omega \right)=n\left(\omega \right)+i\kappa \left(\omega \right)$$

A change in the complex dielectric function under the influence of external conditions leads to a corresponding change in the refractive index n(ω) and the extinction coefficient κ(ω):

(8) $$n\left(\omega \right)=\sqrt{\frac{\sqrt{\varepsilon_{1}^{2}\left(\omega \right)+\varepsilon_{2}^{2}\left(\omega \right)}+\varepsilon_{1}\left(\omega \right)}{2}};\;\kappa \left(\omega \right)=\sqrt{\frac{\sqrt{\varepsilon_{1}^{2}\left(\omega \right)+\varepsilon_{2}^{2}\left(\omega \right)}-\varepsilon_{1}\left(\omega \right)}{2}}$$

The refractive index n(ω) and the extinction coefficient κ(ω) determine the spectral dependences of absorptance A(ω), reflectance R(ω), and transmittance T(ω). These quantities are comparable with experimental data and satisfy the energy conservation [79] R+T+A=1, neglecting scattering losses.

For normal incidence at an interface between air and material and for a single-pass propagation through an absorbing layer of thickness *d*, the reflectance R(ω), the absorptance A(ω) and the transmittance T(ω) have the following form:

(9) $$\begin{array}{cccc} A(\omega ) & =1-e^{-\alpha (\omega )d},\; & \;\alpha (\omega ) & =\frac{2\omega }{c}\kappa \left(\omega \right)=\frac{4\pi }{\lambda }\kappa \left(\frac{2\pi c}{\lambda }\right),\\ R(\omega ) & ={\left|r\left(\omega \right)\right|}^{2}={\left|\frac{n(\omega )-1+i\kappa (\omega )}{n(\omega )+1+i\kappa (\omega )}\right|}^{2},\; & \;T(\omega ) & =\left(1-R\left(\omega \right)\right)e^{-\alpha (\omega )d}. \end{array}$$

Here, r(ω) denotes the Fresnel reflection coefficient, α(ω) is the absorption coefficient. In general, stimulus-induced variations in $\tilde{\varepsilon }\left(\omega \right)$ lead to measurable changes in n(ω) and κ(ω), and consequently R(ω) and T(ω). Therefore, chromic phenomena can be quantitatively analyzed by tracking how $\tilde{\varepsilon }\left(\omega \right)$ evolves with external control parameters.

### 3.2. Drude–Lorentz Model and Effective Medium Approximation

In a phenomenological description, the complex dielectric function and its evolution under an external parameter *x* (temperature, electric field, illumination, etc.) are parameterized by the generalized Drude–Lorentz (DL) model [48]:

(10) $$\tilde{\varepsilon }\left(\omega ,x\right)=\varepsilon_{\infty }-\frac{\omega_{p}^{2}\left(x\right)}{\omega^{2}+i\gamma \left(x\right)\omega }+\sum_{j}\frac{f_{j}\left(x\right)}{\omega_{0j}^{2}\left(x\right)-\omega^{2}-i\Gamma_{j}\left(x\right)\omega }$$

The first term in Equation (10) $\varepsilon_{\infty }$ is the high-frequency optical dielectric constant and represents the contributions from electronic transitions far above the considered spectral window.

The second term in Equation (10) is the Drude contribution and represents the free-carrier response. Here, $\omega_{p}=\sqrt{Ne^{2}/m^{*}\varepsilon_{0}}$ is the plasma frequency, $\gamma =\tau^{-1}$ is the damping coefficient, *N* is the concentration of free carriers, and $\varepsilon_{0}$ is the dielectric permittivity of vacuum. The Drude term becomes pronounced upon entering the metallic phase (or under conditions of sufficiently high free-carrier density), whereas it is negligible in the insulating state.

The last term in Equation (10) is the sum of Lorentz oscillators and represents bound charge excitations (interband transitions, charge transfer bands, phonons, and polaron-related absorption). Here, $\omega_{0j}$ and $\Gamma_{j}$ are the resonance frequency and damping of the *j*-th oscillator, whereas $f_{j}=N_{j}e^{2}/m^{*}\varepsilon_{0}$ is its spectral weight (or the oscillator strength). Unlike the Drude response, Lorentz-type contributions are present in both metallic and insulating phases, although their spectral weights and resonance parameters may change across the transition.

Within the DL model, the real part $\varepsilon_{1}$ and imaginary part $\varepsilon_{2}$ of the dielectric function are expressed as:

(11) $$\begin{array}{c} \varepsilon_{1}=\varepsilon_{\infty }+\sum_{j=1}^{N}f_{j}\frac{\omega_{0j}^{2}-\omega^{2}}{{\left(\omega_{0j}^{2}-\omega^{2}\right)}^{2}+\gamma_{j}^{2}\omega^{2}}-\frac{\omega_{p}^{2}}{\omega^{2}+\gamma_{D}^{2}}\\ \varepsilon_{2}=\sum_{j=1}^{N}f_{j}\frac{\gamma_{j}\omega }{{\left(\omega_{0j}^{2}-\omega^{2}\right)}^{2}+\gamma_{j}^{2}\omega^{2}}-\frac{\omega_{p}^{2}}{\omega^{2}+\gamma_{D}^{2}} \end{array}$$

The Lorentz oscillator model (11) is insufficient to describe the absorption edge and fundamental optical transitions in amorphous (or nanocrystalline) systems. Thus, the Tauc–Lorentz (TL) model is employed to describe the dielectric response. The imaginary part of the dielectric function within the TL formalism is determined by a sum of the corresponding oscillators:

(12) $$\varepsilon_{2}\left(\omega \right)=\sum_{j}\frac{A_{j}\gamma_{j}\omega_{0j}}{\omega }\frac{{\left(\omega -\omega_{g}\right)}^{2}}{{\left(\omega^{2}-\omega_{0j}^{2}\right)}^{2}+\gamma_{j}^{2}\omega^{2}}\Theta \left(\omega -\omega_{g}\right)$$

Here, $A_{j}$ is the TL oscillator strength, $\gamma_{j}$ is the damping coefficient, $\omega_{0j}$ represents the resonance frequencies, $\omega_{g}$ is the Tauc gap, and Θ(x) is the Heaviside step function.

The real part of the dielectric function is obtained from the imaginary part via the Kramers–Kronig relations:

(13) $$\varepsilon_{1}\left(\omega \right)=\varepsilon_{\infty }+\frac{2}{\pi }P\int_{\omega_{g}}^{\infty }\sum_{j}\frac{\xi \;\varepsilon_{2,j}\left(\xi \right)}{\xi^{2}-\omega^{2}}d\xi$$

Here, $\varepsilon_{\infty }$ represents the contribution of high-energy electronic transitions, and P is the Cauchy principal value of the integral.

A common feature of chromic TMOs is the existence of hysteresis, which reflects the presence of two or more (meta)stable states separated by an energy or kinetic barrier. At the variation of an external parameter *x*, the transition between these states occurs asymmetrically, which leads to a difference between the forward $x^{↑}$ and reverse $x^{↓}$ branches and to the formation of a hysteresis loop in the optical constants of the substance. One of the possible ways to model a hysteresis behavior is the Bruggeman Effective Medium Approximation (EMA) [80]. In Bruggeman EMA for a two-phase mixture (spherical inclusion), the effective dielectric function $\tilde{\varepsilon }\left(\omega ,x\right)$ satisfies:

(14) $$f\left(x\right)\frac{\varepsilon_{A}\left(\omega \right)-\tilde{\varepsilon }\left(\omega \right)}{\varepsilon_{A}\left(\omega \right)+2\tilde{\varepsilon }\left(\omega \right)}+\left(1-f\left(x\right)\right)\frac{\varepsilon_{B}\left(\omega \right)-\tilde{\varepsilon }\left(\omega \right)}{\varepsilon_{B}\left(\omega \right)+2\tilde{\varepsilon }\left(\omega \right)}=0$$

Here, $\varepsilon_{A}\left(\omega \right)$ and $\varepsilon_{B}\left(\omega \right)$ are the dielectric functions of phases *A* and *B*, each of which is parameterized by the DL model (10), and f(x) is the volume fraction of phase *A*, which depends on the external parameter *x*. To represent a smooth transition between phases, f(x) can be chosen in a sigmoidal form, e.g.,:

(15) $$f\left(x\right)={\left(1+exp\left(-\frac{x-x_{c}}{\Delta x}\right)\right)}^{-1}$$

Here, $x_{c}$ is the critical value of the external parameter *x* at which optical contrast is observed, and Δx is the sharpness of the transition. Hysteresis can be incorporated by assigning different parameters to the forward and reverse branches, e.g., $x_{c}^{↑}$ upon increasing *x* and $x_{c}^{↓}$ upon decreasing *x*. In this framework, hysteresis becomes directly manifested in $\tilde{\varepsilon }\left(\omega ,x\right)$ and, consequently, in the derived optical spectra n(ω) and κ(ω).

### 3.3. Optical Response Modeling for TMOs

In the following subsections, the Drude–Lorentz (11) and Tauc–Lorentz (13), (12) described above are used to construct schematic illustrations of the optical response of representative chromic TMOs. The model parameters were selected within the ranges reported in the literature for ellipsometry of VO_2_, WO_3_, NiO, and TiO_2_. Therefore, the calculated spectra should not be interpreted as unique fits to a particular experimental sample since optical constants in TMO thin films depend strongly on deposition method, crystallinity, film thickness, grain size, strain state, defect concentration, stoichiometry, and measurement conditions. Instead, the aim of these simulations is to provide a literature-guided phenomenological reconstruction of the characteristic spectral changes associated with different chromic mechanisms. In this sense, the presented spectra illustrate how thermally driven metal–insulator transitions, ion-intercalation-induced polaron formation, and photoinduced polaron states can be represented through changes in the Drude contribution, Lorentz or Tauc–Lorentz oscillators, and effective optical constants.

Using Equations (7)–(12), the complex dielectric function, refractive index, extinction coefficient, absorptance, reflectance, and transmittance are then calculated in order to visualize the optical signatures of each chromic state.

#### 3.3.1. Thermochromism in VO_2_

The optical response of vanadium dioxide is modeled within the DL formalism (10), (11). This approach allows simultaneous description of interband optical transitions and the free-carrier response that emerges across the thermally driven MIT in VO_2_.

The Drude term, defined by the plasma frequency $\omega_{p}$ and damping constant $\gamma_{D}$, accounts for the contribution of free charge carriers present in the metallic phase of VO_2_. In the low-temperature monoclinic insulating phase (M1), realized below the critical temperature $T_{c}\approx 340\;K$, the density of free charge carriers is negligible. As a result, the plasma frequency is equal to zero $\omega_{p}\approx 0$, and the Drude contribution to the dielectric function is absent. In this regime, the optical response of VO_2_ is governed exclusively by Lorentz oscillators. Above the critical temperature $T_{c}$, VO_2_ undergoes an MIT and transitions to the rutile metallic phase (R). As a result, the plasma frequency becomes the non-zero value $\omega_{p}\neq 0$. Consequently, the Drude term becomes active and dominates the low-energy optical response.

Plasma frequency of free charge carriers and collision frequency (damping coefficient) in the Drude contribution to the dielectric function is determined by the optical effective mass $m^{*}$ [81] and the carrier concentration $n_{e}$ [82]:

(16) $$\omega_{p}^{2}=\frac{4\pi n_{e}e^{2}}{m^{*}};\;\gamma_{D}=\frac{e}{\mu_{opt}m^{*}}\;\left(CGS\;units\right)$$

Here, $\mu_{opt}$ is optical mobility. According to Rosevear and Paul [83], the optical effective mass of charge carriers in VO_2_ from the Hall effect experiment is $m^{*}\approx 3.5m_{0}$.

In the low-temperature M1 phase, the carrier concentration is relatively small ($n_{e}\approx 10^{18}$–$10^{19}$ cm^−3^). As a result, the Drude contribution to the dielectric function in the optical range is negligible and, within the phenomenological model, can be set to zero ($\omega_{p}\approx 0$). In the metallic R phase, the effective concentration of conducting carriers increases to values typical for metals ($n_{e}\approx 10^{22}$–$10^{23}$ cm^−3^). This leads to a plasma energy of the order of several eV.

The critical threshold carrier concentration for the overlap of localized electronic states can be obtained from the Mott criterion:

(17) $$n_{c}^{1/3}a_{B}^{*}=0.25,\Rightarrow n_{c}={\left(\frac{0.25}{a_{B}^{*}}\right)}^{3};\;a_{B}=\frac{\varepsilon_{r}}{m^{*}/m_{0}}a_{0};\;a_{0}=0.529\;Å$$

Here, $a_{B}$ is the effective Bohr radius, and $\varepsilon_{r}$ denotes the static dielectric permittivity of VO_2_, which is known to increase significantly near the MIT due to enhanced lattice and electronic polarizability. Experimental studies [84,85,86] report values in the range of approximately $\varepsilon_{r}\approx$ 36–100. By using these parameters, the effective Bohr radius is estimated to be in the range of $a_{B}\approx$ 0.54–1.51 nm, yielding a critical carrier concentration of $n_{c}\approx 4.5\times 10^{18}$–$9.7\times 10^{19}$ cm^−3^. This range is consistent with experimentally observed carrier densities at the onset of metallization in VO_2_. For carrier concentrations and mobility typical of the rutile metallic phase [87] ($n_{e}\approx 10^{21}$–$10^{22}$ cm^−3^ and μ≈ 0.1–0.2 cm^2^/V·s), the resulting plasma energy is of the order of $\hbar \omega_{p}\approx$ 0.6–1.9 eV and the damping constant is on the order of $\hbar \gamma_{D}\approx$ 1.6–3.1 eV.

For the Lorentz contribution, the optical response of VO_2_ was described by using a minimal set of effective oscillators representing the main characteristic bands [82,88,89]:

(i) The low-energy NIR band (∼0.7 eV) is associated with low-energy electronic reconstruction in the monoclinic phase and reflects the Peierls-induced splitting of the V-$3d_{\left|\right|}$ states, with an additional contribution from electronic correlations. In the M1 phase, this band exhibits a relatively large oscillator strength (f∼ 0.4–0.5) and substantial broadening (ℏγ∼ 0.4–0.7 eV), reflecting the presence of gapped low-energy excitations. Upon the transition to the R phase, the spectral weight associated with this oscillator is strongly suppressed (f→0) and largely transferred to the Drude response.

(ii) The visible (VIS) band (∼1.8–2.5 eV) represents the dominant interband transition in the VIS range, primarily O-2p→V-3d charge transfer (CT) transitions, with possible contributions from intra-*d* (d→d) transitions between split 3d subbands. This band remains present in both phases; however, moderate changes in oscillator strength ($f_{M1}(\approx$1.0–1.3$)\to f_{R}(\approx$0.8–1.0) and damping (ℏγ(≈0.8–1.2 eV)→ℏγ(≈1.0–1.4 eV)) are allowed in the rutile phase, reflecting the modified electronic structure and enhanced carrier scattering in the metallic phase.

(iii) The near-UV band (∼3.2–3.8 eV) represents the higher-energy interband transitions, including additional CT processes and the high-energy tails of O-2p→V-3d transitions. The corresponding Lorentz oscillator shows only weak sensitivity to the MIT (f∼ 0.6–0.9, 1.2–1.6 eV).

(iv) The high-energy UV background (∼5–7 eV) represents a broad Lorentz oscillator describing the cumulative contribution of deep interband transitions, such as O-2p→V-3d/4s/4p states. The high-energy UV background is assumed to be nearly phase-independent (f∼ 2–3, ℏγ∼ 3–5 eV) and primarily ensures a physically consistent high-frequency behavior of the dielectric function.

In the present DL model (11), the parameter $\varepsilon_{\infty }$ is treated as an effective high-frequency dielectric constant accounting for electronic polarizability contributions from interband transitions lying beyond the considered spectral window (∼0–10 eV). Therefore, $\varepsilon_{\infty }$ should not be interpreted as the strict ω→∞ limit of the dielectric function, but rather as an effective residual contribution within the finite-range optical model. In practice, typical values of $\varepsilon_{\infty }$ reported for VO_2_ lie in the range $\varepsilon_{\infty }∼$ 4–8 [82,88,89]. Within the present phenomenological model, $\varepsilon_{\infty }$ was assumed to be identical for both the M1 and R phases, reflecting the fact that high-energy interband transitions are only weakly affected by the MIT.

**Figure 4:**
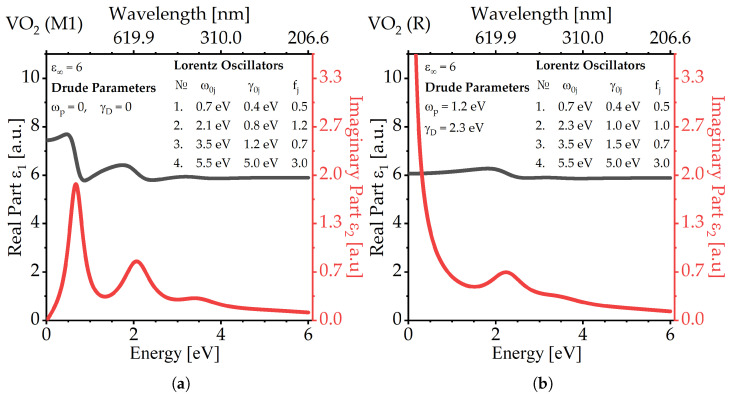
Representative spectra of the real $\varepsilon_{1}$ and imaginary $\varepsilon_{2}$ parts of dielectric function in VO_2_: (**a**) monoclinic vs. (**b**) rutile.

**Figure 5:**
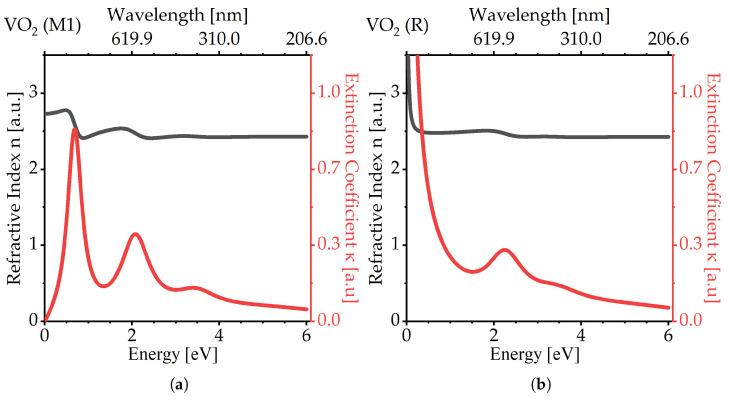
Representative spectra of the refractive index n(ω) and extinction coefficient κ(ω) in VO_2_: (**a**) monoclinic vs. (**b**) rutile.

The evolution of the real and imaginary part of the dielectric function of VO_2_ across the MIT within the present DL model is illustrated in Figure 4. In the M1 phase, the dielectric response is governed only by Lorentz oscillators. On the contrary, in the R phase, the emergence of a pronounced Drude response at low photon energies reflects the contribution of free charge carriers. The refractive index n(ω) and extinction coefficient κ(ω) were calculated from the obtained function within the DL model by using Equation (8). The resulting spectral dependencies across the MIT are shown in Figure 5.

In the VIS range, both phases exhibit comparable values of the refractive index. In particular, at a photon energy of ∼2 eV (corresponding to a wavelength of 619 nm, close to a local maximum in the VIS region), the refractive index is n∼2.5 for both phases. In contrast, the extinction coefficient shows a noticeable phase dependence, with κ∼0.16 in the M1 phase and κ∼0.12 in the R phase, reflecting the redistribution of spectral weight across the MIT.

**Figure 6:**
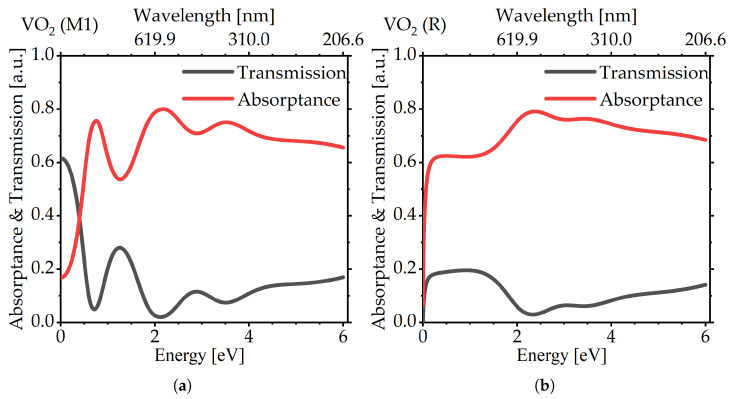
Representative spectra of the transmission T(ω) and absorptance A(ω) for VO_2_: (**a**) monoclinic vs. (**b**) rutile.

By using Equation (9), the spectral dependencies of reflectance, transmittance and absorptance were estimated. The results are shown in Figure 6. As seen across the thermally driven MIT, the low-energy response changes markedly: the metallic phase exhibits a suppressed transmittance and enhanced absorption/reflectance in the NIR, consistent with the emergence of the Drude-like free-carrier contribution.

The literature-guided character of the present VO_2_ parameterization is supported by several spectroscopic ellipsometry and optical-constant studies of monoclinic and rutile VO_2_ thin films. Currie et al. reported temperature-dependent tunability of the complex refractive index of VO_2_, showing a pronounced change in the extinction coefficient during the transition, especially in the near-infrared region [90]. Wan et al. provided broadband optical constants of VO_2_ from the visible to the far-infrared range and demonstrated that, despite sample-to-sample variations, the dominant optical change across the insulator–metal transition is the emergence of a strong metallic low-energy response [91]. Ait Abdelkadir et al. analyzed the temperature-dependent optical properties of undoped and W-doped VO_2_ thin films by spectroscopic ellipsometry, confirming that the transition is reflected in pronounced changes in $\varepsilon_{1}$, $\varepsilon_{2}$, *n*, and κ between the monoclinic and rutile phases [92]. More recent ellipsometric studies by Sun et al. and Morris et al. further emphasize the importance of dispersion model analysis for extracting reliable optical constants of VO_2_ thin films and for describing the temperature-dependent phase transition [93,94]. Overall, these experimental and modeling studies show that the monoclinic phase is characterized by a suppressed free-carrier contribution, whereas the rutile phase develops a pronounced Drude-like response at low photon energies, accompanied by a redistribution of spectral weight and a strong increase in the infrared extinction coefficient. At the same time, the refractive index in the visible range changes more moderately than the infrared optical losses.

#### 3.3.2. Electro- and Gasochromism NiO and WO_3_

Electrochromism and gasochromism in NiO and WO_3_ fundamentally differ from the thermochromic response of VO_2_. Instead of an MIT and a Drude-like free-carrier contribution, the chromic behavior in these materials is governed by the reversible formation of localized polaronic states induced by ion intercalation and deintercalation. Consequently, their optical properties can be described by using a purely Lorentz-type model ($\omega_{p}\to 0$ in (11)), where the appearance and evolution of polaron-related absorption bands dominate the chromic response.

For modeling the electro- and gasochromic properties of NiO within the Lorentz formalism (11), a minimal set of characteristic oscillators is employed as follows [95,96,97,98]:

(i) A polaron-related band in the VIS-NIR region (∼0.8–2.0 eV), associated with the formation of the localized hole polarons arising from the oxidation of Ni^2+^ to Ni^3+^, corresponding to intervalence CT transitions. In the bleached state of NiO, the contribution of this oscillator is negligible, whereas upon deintercalation, its oscillator strength strongly increases and the band becomes significantly broadened.

(ii) A VIS-UV band (∼3.6–4.2 eV), corresponding to the dominant interband transitions in NiO, primarily governed by O-2p→Ni-3d CT processes, with an additional contribution from correlated d→d transitions. The resonance energy of this oscillator remains nearly unchanged upon deintercalation.

(iii) A high-energy UV background (∼6.5–7.5 eV), representing a broad cumulative contribution of deep interband transitions, mainly O-2p→Ni-4s/4p states.

**Figure 7:**
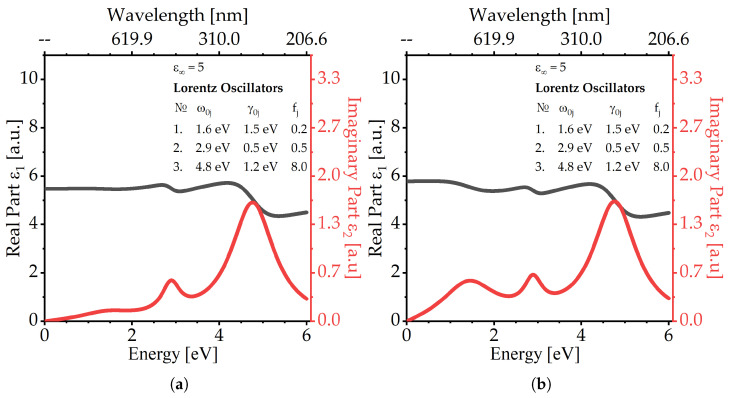
Representative spectra of the real $\varepsilon_{1}$ and imaginary $\varepsilon_{2}$ parts of the dielectric function in NiO: (**a**) pure vs. (**b**) deintercalated.

Based on the present Lorentz model, the real $\varepsilon_{1}$ and imaginary $\varepsilon_{2}$ parts of the dielectric function in NiO were calculated (Figure 7).

As follows from the analysis, the deintercalation process leads to the emergence of an additional absorption band in the VIS-NIR region, which is attributed to polaron-related optical transition.

**Figure 8:**
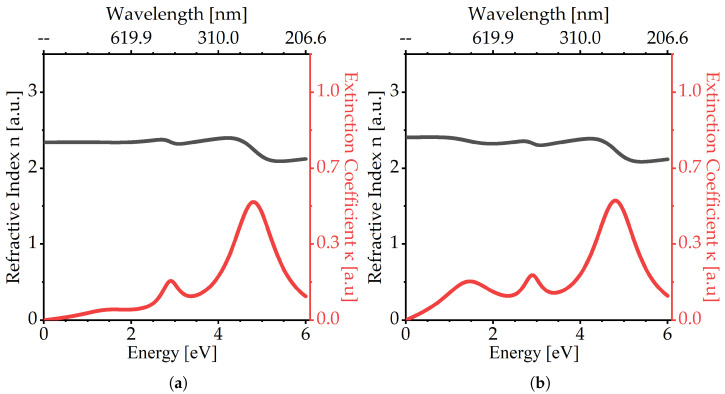
Representative spectra of the refractive index n(ω) and extinction coefficient κ(ω) in NiO: (**a**) pure vs. (**b**) deintercalated.

By using the obtained dielectric function and Equation (8), the refractive index n(ω) and extinction coefficient κ(ω) in NiO were evaluated (Figure 8).

**Figure 9:**
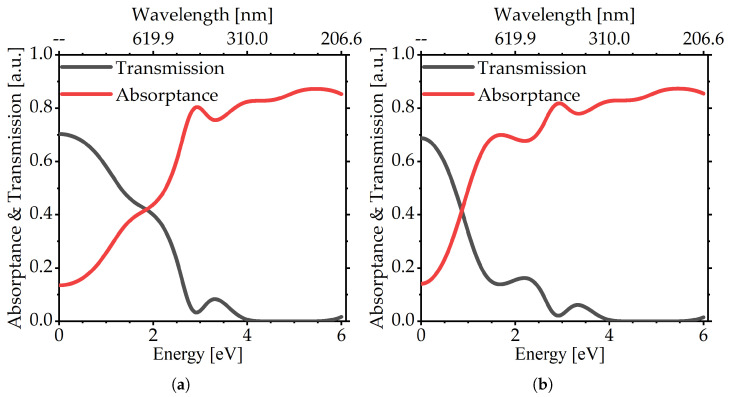
Representative spectra of the transmission T(ω) and absorptance A(ω) for NiO: (**a**) pure vs. (**b**) deintercalated.

By using Equation (9), the spectral dependencies of reflectance, transmittance, and absorptance were estimated. The results are shown in Figure 9. Deintercalation leads to a pronounced increase in absorption in the VIS–NIR range (∼0.5–2.5 eV), accompanied by a strong suppression of transmittance, which is consistent with the emergence of additional polaron-related (sub-gap/IVCT) absorption channels responsible for coloration.

Analogously, to model the chromic response of WO_3_ within the Lorentz formalism, a minimal set of effective oscillators was used [99,100,101]:

(i) A polaron-related VIS-NIR band (∼0.6–1.8 eV), which is associated with the formation of localized electron small polarons upon intercalation-induced reduction of tungsten ions (W^6+^→ W^5+^), corresponding to intervalence CT transitions. Stoichiometric WO_3_ is optically bleached, whereas intercalation results in coloration due to the appearance of additional polaronic absorption channels. Thus, in the bleached state, the contribution of this oscillator is negligible (f→0), while upon intercalation, its oscillator increases and the band becomes significantly broadened.

(ii) A VIS-NIR band (∼2.6–3.2 eV), which represents the dominant interband transitions in WO_3_ (CT process O-2p→W-5d). The resonance energy of this oscillator remains nearly unchanged upon intercalation.

(iii) A high-energy UV background (∼5–7 eV), which corresponds to a broad cumulative contribution of deep interband transitions (O-2p→W-5d/6s/6p). This high-energy contribution is assumed to be only weakly affected by the intercalation process within the considered spectral window (∼0–10 eV).

**Figure 10:**
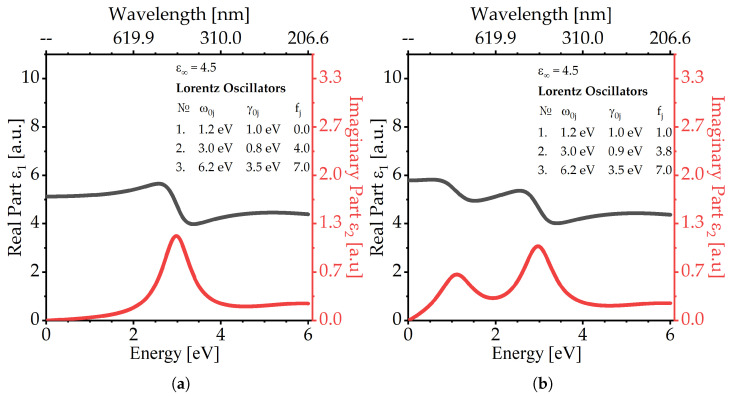
Representative spectra of the real $\varepsilon_{1}$ and imaginary $\varepsilon_{2}$ parts of the dielectric function in WO_3_: (**a**) pure vs. (**b**) intercalated.

Based on the present Lorentz model, the real $\varepsilon_{1}$ and imaginary $\varepsilon_{2}$ parts of the dielectric function in WO_3_ were calculated (Figure 10).

**Figure 11:**
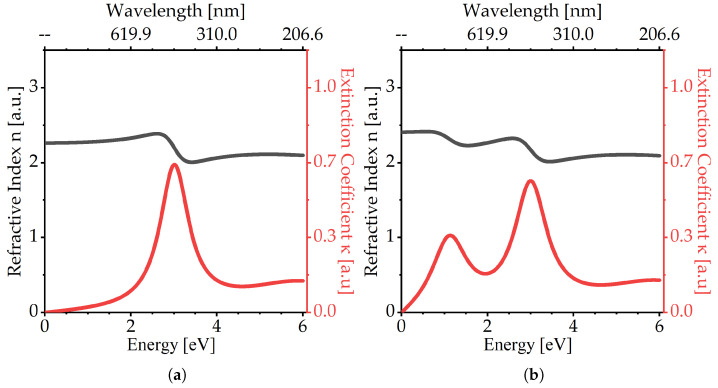
Representative spectra of the refractive index n(ω) and extinction coefficient κ(ω) in WO_3_: (**a**) pure vs. (**b**) intercalated.

Using the obtained dielectric function and Equation (8), the refractive index n(ω) and extinction coefficient κ(ω) in WO_3_ were evaluated (Figure 11).

At the photon energy corresponding to the local maximum in the VIS range (ℏω≈ 2.6 eV, λ≈ 477 nm), pure WO_3_ is characterized by n≈2.4 and κ≈0.20. Upon intercalation, the refractive index decreases to n≈2.3, whereas the extinction coefficient increases to κ≈0.21, reflecting enhanced optical absorption associated with the polaron-related contribution.

**Figure 12:**
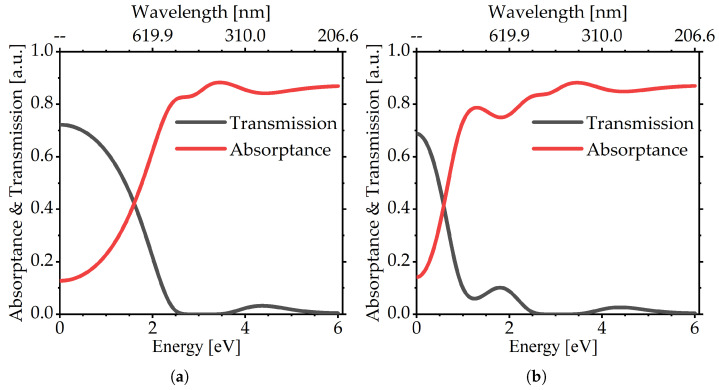
Representative spectra of the transmission T(ω) and absorptance A(ω) for WO_3_: (**a**) pure vs. (**b**) intercalated.

By using Equation (9), the spectral dependencies of reflectance, transmittance, and absorptance were estimated. The results are shown in Figure 12. Intercalation results in a pronounced enhancement in absorption in the VIS–NIR range (∼0.5–2.5 eV) accompanied by a strong suppression of transmittance, which is consistent with the appearance of electron polaron/IVCT absorption.

The literature-guided character of the present Lorentz parameterization for NiO and WO_3_ is supported by optical-constant and spectroscopic ellipsometry studies of these electrochromic oxides. For NiO, the classical optical analysis by Powell and Spicer showed that the complex refractive index and dielectric function are dominated by strong interband and CT features in the near-UV and UV spectral ranges, providing the background dielectric response of the material [95]. Spectroscopic ellipsometry characterization of sputtered electrochromic WO_3_ and NiO films further demonstrated that their optical constants can be represented using oscillator-based dispersion models, while also emphasizing the sensitivity of the extracted parameters to film morphology, optical inhomogeneity, and preparation conditions [102]. For WO_3_, ellipsometric and transmission-based studies of thin films and WO_3_-based functional nanostructures show that the refractive index and extinction coefficient depend strongly on microstructure, stoichiometry, and the presence of catalytic or metallic layers relevant for gasochromic hydrogen sensing [103]. More recent full-spectrum electrochromic ellipsometry of WO_3_ with different Li^+^ insertion levels further demonstrates that increasing intercalation produces a systematic enhancement in absorption and a corresponding reduction in transmittance, consistent with W^5+^/W^6+^ intervalence CT and electron polaron absorption [104].

#### 3.3.3. Photochromism in TiO_2_

Photochromism in amorphous TiO_2_ is governed by a mechanism similar to intercalation–deintercalation processes in WO_3_-NiO systems. However, in the case of photochromism, no external reservoir of charge carriers is involved, and the polaronic states are generated directly by optical excitation. The Lorentz oscillator model (11) is insufficient to describe the absorption edge and fundamental optical transitions in amorphous (or nanocrystalline) systems. Thus, the Tauc–Lorentz model (12), (13) is employed to describe the dielectric response.

Analogously to WO_3_ and NiO, the minimal set of characteristic TL oscillators for modeling the chromic properties of TiO_2_ consists of the following bands:

(i) A polaron-related VIS-NIR band (∼0.8–1.6 eV), which is associated with the formation of localized small photopolarons induced by photoexcitation and accompanied by changes in the titanium valence state (Ti^4+^→Ti^3+^). Thus, in the initial bleached state, the contribution of this oscillator is negligible (f→0), while upon photoexcitation, its oscillator strength increases significantly and the band becomes broadened.

(ii) A VIS-NIR band (∼3–4 eV), which represents the dominant interband transitions in TiO_2_ (CT process O-2p→Ti-3d). The resonance energy of this oscillator remains nearly unchanged upon polaron photoexcitation.

(iii) A high-energy UV background (∼5–10 eV), which corresponds to a broad cumulative contribution of deeper interband transitions (O-2p→Ti-3d/4s). This high-energy contribution is assumed to be only weakly affected by the polaron excitation process within the considered spectral window (∼0–10 eV).

The Tauc gap $\omega_{g}$ represents the effective optical bandgap of the amorphous TiO_2_ matrix, defining the onset of interband absorption. In contrast to the fundamental bandgap of crystalline TiO_2_, the Tauc gap accounts for disorder-induced band tailing and localized states. Typical values of Tauc gap for a-TiO_2_ lie in the range $\omega_{g}∼$ 3.2–3.6 eV.

**Figure 13:**
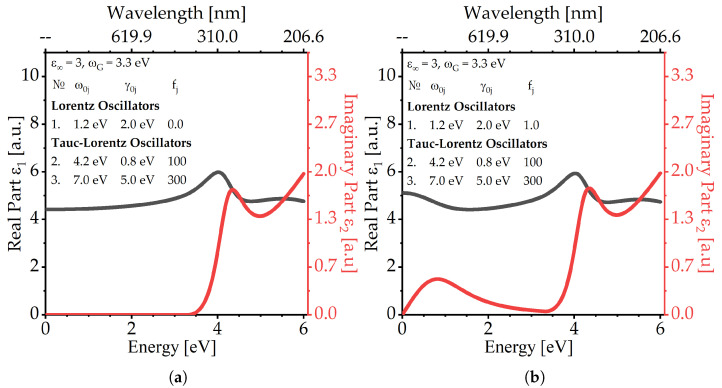
Representative spectra of the real $\varepsilon_{1}$ and imaginary $\varepsilon_{2}$ parts of the dielectric function in TiO_2_: (**a**) initial vs. (**b**) photoexcited.

Based on the present Tauc–Lorentz model, the real $\varepsilon_{1}$ and imaginary $\varepsilon_{2}$ parts of the dielectric function in TiO_2_ were calculated (Figure 13).

**Figure 14:**
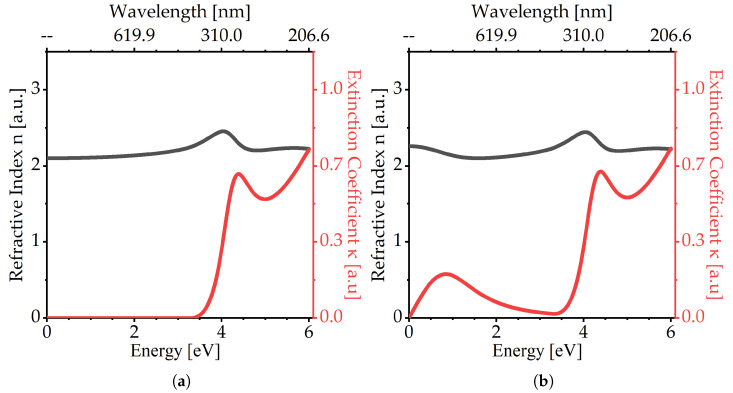
Representative spectra of the refractive index n(ω) and extinction coefficient κ(ω) in TiO_2_: (**a**) initial vs. (**b**) photoexcited.

Using the obtained dielectric function and Equation (8), the refractive index n(ω) and extinction coefficient κ(ω) in TiO_2_ were evaluated (Figure 14).

**Figure 15:**
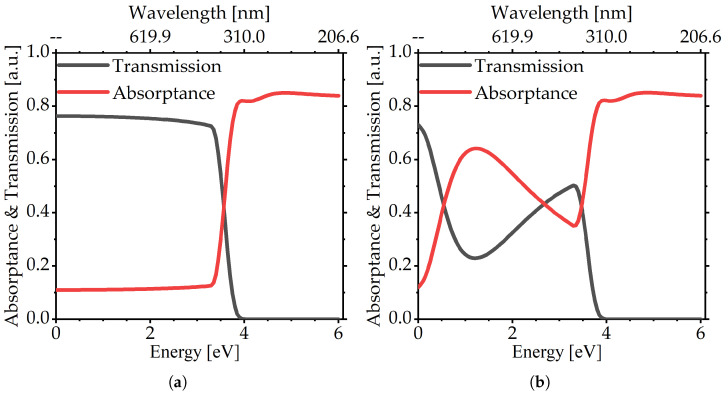
Representative spectra of the transmission T(ω) and absorptance A(ω) for TiO_2_: (**a**) initial vs. (**b**) photoexcited.

By using Equation (9), the spectral dependencies of reflectance, transmittance, and absorptance were estimated. The results are shown in Figure 15.

The literature-guided character of the present TL parameterization for TiO_2_ is supported by optical-constant, ellipsometric, and photochromic studies of amorphous or nanostructured TiO_2_ systems. Spectrophotometric and spectroscopic ellipsometry analyses of amorphous TiO_2_ thin films show that their optical response is dominated by a UV absorption edge with a band gap of the order of 3.3 eV, while the refractive index, extinction coefficient, and real and imaginary parts of the dielectric function can be consistently described using amorphous dispersion models [105]. This behavior justifies the use of a TL description for the initial, non-irradiated state. Under UV illumination, amorphous TiO_2_ nanotube arrays exhibit a progressive photochromic response, manifested by visible darkening and an increase in the absorption intensity in the 400–700 nm range with irradiation time [106]. Reversible photodoping studies of TiO_2_ nanoparticles further show that UV irradiation can strongly reduce optical transmittance and enhance absorption from the visible to the near-infrared region, while exposure to air restores the initial high-transmittance state [107]. These observations are consistent with the formation of photoinduced trapped carriers, Ti^3+^ centers, oxygen-vacancy-related states, and small-polaron-like absorption channels.

### 3.4. Scope and Limitations of the Phenomenological Modeling

The DL and TL approaches used in this work provide a compact phenomenological framework for representing the optical response of chromic TMOs. Their main advantage is that they allow the complex dielectric function to be decomposed into physically interpretable contributions, such as free-carrier response, interband transitions, absorption by localized states, and effective phase mixing. In this sense, these models are useful for connecting experimentally accessible optical quantities, such as ε(ω), n(ω), κ(ω), T(ω), and A(ω), with the dominant chromic mechanism. However, these models also have important limitations.

The DL model does not explicitly describe electronic correlations, electron–phonon coupling, lattice reconstruction, or defect formation. In strongly correlated systems such as VO_2_, the Drude term should therefore be regarded as an effective representation of the metallic free-carrier response rather than as a complete microscopic description of the Peierls–Mott transition. Similarly, Lorentz oscillators used for WO_3_ and NiO represent effective absorption channels associated with interband transitions, mixed-valence states, and polaronic absorption, but their parameters are not unique and may depend on sample preparation, crystallinity, stoichiometry, morphology, and measurement conditions.

The TL model is particularly useful for amorphous or nanocrystalline oxides, where the optical absorption edge is influenced by disorder and band-tail states. Nevertheless, in systems such as TiO_2_, this model does not uniquely distinguish between different microscopic origins of subgap absorption, such as oxygen vacancies, Ti^3+^ centers, trapped carriers, or photoinduced small polarons. Therefore, the additional low-energy oscillator introduced in the photoexcited state should be interpreted as an effective optical representation of defect- and polaron-related absorption rather than as a unique microscopic transition.

These limitations highlight the importance of combining phenomenological optical modeling with microscopic interpretation. Phenomenological models describe how spectra change, whereas microscopic models explain why the corresponding optical parameters change in response to external stimuli. For this reason, the chromic response of TMOs is most consistently understood by linking microscopic mechanisms, such as Peierls–Mott transitions, ion-intercalation-driven polaron formation, and photoinduced defect states, with their effective signatures in the dielectric function.

The main microscopic mechanisms, external stimuli, phenomenological optical signatures, representative applications, and practical limitations of the selected benchmark TMOs are summarized in Table 2. This comparison highlights how different chromic responses can be represented within the same phenomenological optical framework through changes in the Drude contribution, Lorentz oscillators, Tauc–Lorentz absorption edge, and effective extinction coefficient.

## 4. Conclusions

Chromism in TMOs emerges as a multiscale phenomenon linking microscopic electronic mechanisms with macroscopic optical response and device functionality. As shown in this review, two principal microscopic origins govern chromic behavior: phase-transition-driven and polaron-injection-driven mechanisms. In the first case, chromism arises from a reconstruction of the electronic structure associated with a Peierls–Mott MIT, typically controlled by temperature, leading to thermochromism. In the second case, chromic response is governed by the formation and modulation of polaronic states, which introduce localized energy levels and additional absorption channels. Depending on the excitation conditions, this mechanism manifests as electrochromism, gasochromism, or photochromism.

From a phenomenological perspective, these mechanisms produce distinct optical signatures. Phase-transition-driven systems, such as VO_2_, are characterized by the emergence or suppression of a Drude-like free-carrier response, leading to strong modulation in the near-infrared region. In contrast, polaron-based systems, including WO_3_, NiO, and TiO_2_, exhibit additional VIS/NIR absorption bands associated with localized states. These effects can be effectively described using Drude–Lorentz-type models, which, although not microscopic, provide a practical framework for interpreting experimental spectra and linking optical properties to underlying physical processes.

Overall, the results demonstrate that chromism in TMOs cannot be reduced to a single universal mechanism but instead reflects different forms of electronic-structure reorganization. The balance between optical contrast, controllability, and stability depends on the dominant mechanism and material system. Future progress will rely on combining microscopic understanding with materials engineering strategies, including defect control, doping, and nanostructuring, as well as on developing more consistent links between theoretical models and experimental optical response.

## Figures and Tables

**Table 1:** Summary of chromism mechanisms in transition-metal oxides, linking microscopic origin and external control parameters.

Chromism Type	Thermochromism	Electrochromism	Gasochromism	Photochromism
Microscopic mechanism	Peierls–Mott MIT	Small-polaron injection	Redox-driven polaron formation	Photoinduced polaron formation
Control parameter	Temperature (*T*)	Electric field/voltage	Gas concentration (e.g., H_2_)	Light intensity/photon energy
Optical signature	Emergence or suppression of Drude peak	Additional absorption channels	Additional absorption channels	Additional absorption channels
Representative TMOs	VO_2_, V_2_O_3_	WO_3_, NiO	H_x_WO_3_	TiO_2_, WO_3_

**Table 2:** Summary of benchmark chromic TMOs linking microscopic mechanisms, phenomenological optical response, and practical relevance.

TMOs	VO_2_	NiO	WO_3_	TiO_2_
Chromism Type	Thermochromism	Electrochromism	Electrochromism	Photochromism
	–	–	Gasochromism	–
External Stimuli	Temperature	External field	External field	Light irradiation
	$T_{c}$ = 68 °C	Voltage	Gas concentration	Photon energy
Microscopic Mechanism	V–V Dimerization and Electronic Band-Stucture Reconstruction	Hole small-polaron formation	Electron small-polaron formation	Photopolaron formation
	Peierls–Mott MIT	Deintercalation (e.g., Li ions)	Intercalation or redox processes	Photoinduced charge trapping
	Interplay between U/W and λ	Ni^2+^/Ni^3+^ mixed-valence states	W^6+^/W^5+^ mixed-valence states	Ti^3+^ centers and oxygen vacancies
Phenomenological Optical Signature	Emergence or suppression of the Drude contribution	Additional Lorentz oscillators	Additional Lorentz oscillators	Tauc–Lorentz edge with additional low-energy oscillator
	Redistribution of spectral weight	Effective hole polaron absorption	Effective electron polaron/IVCT absorption	Defect- and polaron-related subgap absorption
	Strong modulation of κ across $T_{c}$ in IR-NIR range	Increase in κ in VIS-NIR range	Increase in κ in VIS-NIR range	Increase in κ in VIS range/subgap region
Representative Applications	Thermochromic smart windows	Complementary anodic layer in electrochromic devices	Electrochromic smart windows	Photochromic coatings
	Optical switching and NIR modulation	WO_3_-based smart window devices	Hydrogen-sensitive optical devices	UV-responsive layers and sensors
Main Limitations	Transition temperature, hysteresis width	Charge balance and degradation	Ion diffusion and switching speed	Weak visible contrast and defect control
	Visible transparency and durability	Stoichiometry and Ni vacancy concentration	Electrolyte stability and cycling durability	Reversibility and long-term stability

## Data Availability

No new data were created or analyzed in this study. Data sharing is not applicable to this article.

## Abbreviations

The following abbreviations are used in this manuscript:

TMO	Transition-metal oxide
MIT	Metal–insulator transition
DL	Drude–Lorentz
TL	Tauc–Lorentz
DMFT	Dynamical mean-field theory
DOS	Density of states
LHB	Lower Hubbard band
UHB	Upper Hubbard band
CB	Conduction band
VB	Valence band
VIS	Visible
NIR	Near-infrared
UV	Ultraviolet
EMA	Effective medium approximation
CT	Charge transfer

## References

[1] Yan D., Wang Z., Zhang Z. (2022). Stimuli-Responsive Crystalline Smart Materials: From Rational Design and Fabrication to Applications. Acc. Chem. Res..

[2] Stuart M.A.C., Huck W.T.S., Genzer J., Müller M., Ober C., Stamm M., Sukhorukov G.B., Szleifer I., Tsukruk V.V., Urban M. (2010). Emerging Applications of Stimuli-Responsive Polymer Materials. Nat. Mater..

[3] Imato K., Ooyama Y. (2024). Stimuli-Responsive Smart Polymers Based on Functional Dyes. Polym. J..

[4] Wu S., Sun H., Duan M., Mao H., Wu Y., Zhao H., Lin B. (2023). Applications of Thermochromic and Electrochromic Smart Windows: Materials to Buildings. Cell Rep. Phys. Sci..

[5] Seok H., Son S., Cho J., Choi S., Park K., Kim C., Jeon N., Kim T., Kim H.U. (2022). Chromism-Integrated Sensors and Devices for Visual Indicators. Sensors.

[6] Fu H., Zhang L., Dong Y., Zhang C., Li W. (2023). Recent Advances in Electrochromic Materials and Devices for Camouflage Applications. Mater. Chem. Front..

[7] Yu X., Marks T.J., Facchetti A. (2016). Metal Oxides for Optoelectronic Applications. Nat. Mater..

[8] Rao C.N.R. (1989). Transition Metal Oxides. Annu. Rev. Phys. Chem..

[9] Ahn C., Cavalleri A., Georges A., Ismail-Beigi S., Millis A.J., Triscone J.M. (2021). Designing and Controlling the Properties of Transition Metal Oxide Quantum Materials. Nat. Mater..

[10] Ganduglia-Pirovano M.V., Hofmann A., Sauer J. (2007). Oxygen Vacancies in Transition Metal and Rare Earth Oxides: Current State of Understanding and Remaining Challenges. Surf. Sci. Rep..

[11] Granqvist C.G. (2014). Electrochromics for Smart Windows: Oxide-based Thin Films and Devices. Thin Solid Film..

[12] Haverkort M.W., Hu Z., Tanaka A., Reichelt W., Streltsov S.V., Korotin M.A., Anisimov V.I., Hsieh H.H., Lin H.J., Chen C.T. (2005). Orbital-Assisted Metal-Insulator Transition in VO_2_. Phys. Rev. Lett..

[13] Zylbersztejn A., Mott N.F. (1975). Metal-Insulator Transition in Vanadium Dioxide. Phys. Rev. B.

[14] Park J.H., Coy J.M., Kasirga T.S., Huang C., Fei Z., Hunter S., Cobden D.H. (2013). Measurement of a Solid-State Triple Point at the Metal–Insulator Transition in VO_2_. Nature.

[15] Granqvist C.G. (2002). Handbook of Inorganic Electrochomic Materials.

[16] Ping Y., Rocca D., Galli G. (2013). Optical Properties of Tungsten Trioxide from First-Principles Calculations. Phys. Rev. B.

[17] Fujimori A., Minami F. (1984). Valence-Band Photoemission and Optical Absorption in Nickel Compounds. Phys. Rev. B.

[18] Mo S.D., Ching W.Y. (1995). Electronic and Optical Properties of Three Phases of Titanium Dioxide: Rutile, Anatase, and Brookite. Phys. Rev. B.

[19] Park J.H., Tjeng L.H., Tanaka A., Allen J.W., Chen C.T., Metcalf P., Honig J.M., de Groot F.M.F., Sawatzky G.A. (2000). Spin and Orbital Occupation and Phase Transitions in V_2_O_3_. Phys. Rev. B.

[20] Rodolakis F., Hansmann P., Rueff J.P., Toschi A., Haverkort M.W., Sangiovanni G., Tanaka A., Saha-Dasgupta T., Andersen O.K., Held K. (2010). Inequivalent Routes across the Mott Transition in V_2_O_3_ Explored by X-Ray Absorption. Phys. Rev. Lett..

[21] McWhan D.B., Menth A., Remeika J.P., Brinkman W.F., Rice T.M. (1973). Metal-Insulator Transitions in Pure and Doped V_2_O_3_. Phys. Rev. B.

[22] Parker J.C., Lam D.J., Xu Y.N., Ching W.Y. (1990). Optical Properties of Vanadium Pentoxide Determined from Ellipsometry and Band-Structure Calculations. Phys. Rev. B.

[23] Ho Y.K., Chang C.C., Wei D.H., Dong C.L., Chen C.L., Chen J.L., Jang W.L., Hsu C.C., Chan T.S., Kumar K. (2013). Characterization of Gasochromic Vanadium Oxides Films by X-ray Absorption Spectroscopy. Thin Solid Film..

[24] Chen C.L., Dong C.L., Ho Y.K., Chang C.C., Wei D.H., Chan T.C., Chen J.L., Jang W.L., Hsu C.C., Kumar K. (2013). Electronic and Atomic Structures of Gasochromic V_2_O_5_ Films. Europhys. Lett..

[25] Mirzaei A., Kim J.H., Kim H.W., Kim S.S. (2019). Gasochromic WO_3_ Nanostructures for the Detection of Hydrogen Gas: An Overview. Appl. Sci..

[26] da Silva Júnior M.G., Arzuza L.C.C., Sales H.B., Farias R.M.d.C., Neves G.d.A., Lira H.d.L., Menezes R.R. (2023). A Brief Review of MoO_3_ and MoO_3_-Based Materials and Recent Technological Applications in Gas Sensors, Lithium-Ion Batteries, Adsorption, and Photocatalysis. Materials.

[27] Pang R., Wang Z., Li J., Chen K. (2023). Polymorphs of Nb_2_O_5_ Compound and Their Electrical Energy Storage Applications. Materials.

[28] Zhang Z., Zhang L., Zhou Y., Cui Y., Chen Z., Liu Y., Li J., Long Y., Gao Y. (2023). Thermochromic Energy Efficient Windows: Fundamentals, Recent Advances, and Perspectives. Chem. Rev..

[29] Liu M., Li X., Zhang W., Li L., Li L., Wang C., Pei G., Zhao B., Zou C. (2025). Three-State Thermochromic Smart Window for Building Energy-Saving. Adv. Sci..

[30] Cui Y., Ke Y., Liu C., Chen Z., Wang N., Zhang L., Zhou Y., Wang S., Gao Y., Long Y. (2018). Thermochromic VO_2_ for Energy-Efficient Smart Windows. Joule.

[31] Romanyuk A., Steiner R., Marot L., Oelhafen P. (2007). Temperature-Induced Metal–Semiconductor Transition in W-doped VO_2_ Films Studied by Photoelectron Spectroscopy. Sol. Energy Mater. Sol. Cells.

[32] Chen S., Dai L., Liu J., Gao Y., Liu X., Chen Z., Zhou J., Cao C., Han P., Luo H. (2013). The Visible Transmittance and Solar Modulation Ability of VO_2_ Flexible Foils Simultaneously Improved by Ti Doping: An Optimization and First Principle Study. Phys. Chem. Chem. Phys..

[33] Mlyuka N.R., Niklasson G.A., Granqvist C.G. (2009). Mg Doping of Thermochromic VO_2_ Films Enhances the Optical Transmittance and Decreases the Metal-Insulator Transition Temperature. Appl. Phys. Lett..

[34] Binions R., Piccirillo C., Parkin I.P. (2007). Tungsten Doped Vanadium Dioxide Thin Films Prepared by Atmospheric Pressure Chemical Vapour Deposition from Vanadyl Acetylacetonate and Tungsten Hexachloride. Surf. Coat. Technol..

[35] Vlček J., Kaufman M., Mohammadi Nia E., Houška J., Jiang J., Čerstvý R., Haviar S., Meletis E.I. (2026). High-Performance Thermochromic Multilayer Coatings of W-Doped VO_2_ Nanoparticles Dispersed in an SiO_2_ Matrix Prepared on Glass at a Low Temperature. ACS Appl. Nano Mater..

[36] Becker M., Wollenweber-Bienerth Y.R., Hartmann S., Polity A., Chatterjee S., Klar P.J. (2025). Optimization and Validation of the Thermochromic Performance of a Trilayer Coating of TiO_2_/VO_2_/TiO_2_ for Smart Windows. ACS Appl. Electron. Mater..

[37] Liu H., Zong H., Yan L., Zhou D., Yin Y., Cao G., Bian L., Kang C., Li M. (2021). SnO_2_/VO_2_/SnO_2_ Tri-Layer Thermochromic Films with High Luminous Transmittance, Remarkable Solar Modulation Ability and Excellent Hydrophobicity Grown on Glass Substrates. Infrared Phys. Technol..

[38] Strelcov E., Lilach Y., Kolmakov A. (2009). Gas Sensor Based on Metal-Insulator Transition in VO_2_ Nanowire Thermistor. Nano Lett..

[39] Chen J.L., Chang C.C., Ho Y.K., Chen C.L., Hsu C.C., Jang W.L., Wei D.H., Dong C.L., Pao C.W., Lee J.F. (2015). Behind the Color Switching in Gasochromic VO_2_. Phys. Chem. Chem. Phys..

[40] Zhang J., Tu J.P., Xia X.H., Qiao Y., Lu Y. (2009). An All-Solid-State Electrochromic Device Based on NiO/WO_3_ Complementary Structure and Solid Hybrid Polyelectrolyte. Sol. Energy Mater. Sol. Cells.

[41] Au B.W.C., Chan K.Y. (2022). Towards an All-Solid-State Electrochromic Device: A Review of Solid-State Electrolytes and the Way Forward. Polymers.

[42] Ding Y., Wang M., Mei Z., Diao X. (2022). Different Ion-Based Electrolytes for Electrochromic Devices: A Review. Sol. Energy Mater. Sol. Cells.

[43] Guo J., Liang Y., Zhang S., Ma D., Yang T., Zhang W., Li H., Cao S., Zou B. (2023). Recent Progress in Improving Strategies of Metal Oxide-Based Electrochromic Smart Window. Green Energy Resour..

[44] Imada M., Fujimori A., Tokura Y. (1998). Metal-Insulator Transitions. Rev. Mod. Phys..

[45] Millis A.J., Baeriswyl D., Degiorgi L. (2004). Optical Conductivity and Correlated Electron Physics. Strong Interactions in Low Dimensions.

[46] Reticcioli M., Diebold U., Kresse G., Franchini C. (2020). Small Polarons in Transition Metal Oxides. Handbook of Materials Modeling.

[47] Lany S. (2015). Semiconducting Transition Metal Oxides. J. Phys. Condens. Matter.

[48] Wooten F. (1972). Optical Properties of Solids.

[49] Mott N.F. (1949). The Basis of the Electron Theory of Metals, with Special Reference to the Transition Metals. Proc. Phys. Soc. Sect. A.

[50] Mott N.F. (1968). Metal-Insulator Transition. Rev. Mod. Phys..

[51] Hubbard J. (1965). Electron Correlations in Narrow Energy Bands—IV. The Atomic Representation. Proc. R. Soc. Lond. A Math. Phys. Sci..

[52] Kemeny G. (1965). A Model of the Insulator-Metal Transition Part I. Two-body Correlations. Ann. Phys..

[53] Gutzwiller M.C. (1965). Correlation of Electrons in a Narrow *s* Band. Phys. Rev..

[54] Brinkman W.F., Rice T.M. (1970). Single-Particle Excitations in Magnetic Insulators. Phys. Rev. B.

[55] Metzner W., Vollhardt D. (1989). Correlated Lattice Fermions in *d* = *∞* Dimensions. Phys. Rev. Lett..

[56] Georges A., Kotliar G., Krauth W., Rozenberg M.J. (1996). Dynamical Mean-Field Theory of Strongly Correlated Fermion Systems and the Limit of Infinite Dimensions. Rev. Mod. Phys..

[57] Kotliar G., Savrasov S.Y., Haule K., Oudovenko V.S., Parcollet O., Marianetti C.A. (2006). Electronic Structure Calculations with Dynamical Mean-Field Theory. Rev. Mod. Phys..

[58] Peierls R.E. (1955). Quantum Theory of Solids.

[59] Kohn W. (1959). Image of the Fermi Surface in the Vibration Spectrum of a Metal. Phys. Rev. Lett..

[60] Fröhlich H. (1954). On the Theory of Superconductivity: The One-Dimensional Case. Proc. R. Soc. Lond. A Math. Phys. Sci..

[61] Grüner G. (1994). Density Waves in Solids.

[62] Grüner G. (1988). The Dynamics of Charge-Density Waves. Rev. Mod. Phys..

[63] Biermann S. (2014). Dynamical Screening Effects in Correlated Electron Materials-a Progress Report on Combined Many-Body Perturbation and Dynamical Mean Field Theory: ‘GW + DMFT’. J. Phys. Condens. Matter Inst. Phys. J..

[64] Giustino F. (2017). Electron-Phonon Interactions from First Principles. Rev. Mod. Phys..

[65] Holstein T. (1959). Studies of Polaron Motion: Part I. The Molecular-Crystal Model. Ann. Phys..

[66] Capone M., Sangiovanni G., Castellani C., Di Castro C., Grilli M. (2004). Phase Separation Close to the Density-Driven Mott Transition in the Hubbard-Holstein Model. Phys. Rev. Lett..

[67] Emin D. (2012). Polarons.

[68] Landau L. (1933). Über Die Bewegung Der Elektronen in Kristallgitter. Phys. Z. Sowjetunion.

[69] Pekar S.I. (1948). Quantum States and Optical Transitions of Electron in a Polaron and at a Color Center of a Crystal. Zh. Eksp. Teor. Fiz..

[70] Landau L., Pekar S.I. (1965). The Effective Mass of Polaron. Collected Papers of L.D. Landau.

[71] Fröhlich H., Pelzer H., Zienau S. (1950). XX. Properties of Slow Electrons in Polar Materials. Lond. Edinb. Dublin Philos. Mag. J. Sci..

[72] Fröhlich H. (1954). Electrons in Lattice Fields. Adv. Phys..

[73] Holstein T. (2000). Studies of Polaron Motion: Part II. The “Small” Polaron. Ann. Phys..

[74] Hébert F., Xiao B., Rousseau V.G., Scalettar R.T., Batrouni G.G. (2019). One-Dimensional Hubbard-Holstein Model with Finite-Range Electron-Phonon Coupling. Phys. Rev. B.

[75] Atkins P., de Paula J., Keeler J., Atkins P., de Paula J., Keeler J. (2022). Atkins’ Physical Chemistry.

[76] Deb S.K. (1969). A Novel Electrophotographic System. Appl. Opt..

[77] Faughnan B.W., Crandall R.S., Heyman P.M. (1975). Electrochromism in WO_3_ Amorphous Films. RCA Rev..

[78] Emin D. (1993). Optical Properties of Large and Small Polarons and Bipolarons. Phys. Rev. B.

[79] Born M., Wolf E. (1999). Principles of Optics: Electromagnetic Theory of Propagation, Interference and Diffraction of Light.

[80] Bergman D.J., Stroud D. (1992). Physical Properties of Macroscopically Inhomogeneous Media. Solid State Phys..

[81] Cohen M.H. (1958). Optical Constants, Heat Capacity and the Fermi Surface. Philos. Mag. J. Theor. Exp. Appl. Phys..

[82] Verleur H.W., Barker A.S., Berglund C.N. (1968). Optical Properties of VO_2_ between 0.25 and 5 eV. Phys. Rev..

[83] Rosevear W.H., Paul W. (1973). Hall Effect in VO_2_ near the Semiconductor-to-Metal Transition. Phys. Rev. B.

[84] Mansingh A., Singh R., Sayer M. (1978). Dielectric Behaviour of Vanadium Dioxide. Phys. Status Solidi (a).

[85] Yang Z., Ko C., Balakrishnan V., Gopalakrishnan G., Ramanathan S. (2010). Dielectric and Carrier Transport Properties of Vanadium Dioxide Thin Films across the Phase Transition Utilizing Gated Capacitor Devices. Phys. Rev. B.

[86] Paik H., Moyer J.A., Spila T., Tashman J.W., Mundy J.A., Freeman E., Shukla N., Lapano J.M., Engel-Herbert R., Zander W. (2015). Transport Properties of Ultra-Thin VO_2_ Films on (001) TiO_2_ Grown by Reactive Molecular-Beam Epitaxy. Appl. Phys. Lett..

[87] Inomata N., Usuda T., Yamamoto Y., H. Zoellner M., Costina I., Ono T. (2022). Effects of Temperature and Doping Concentration on the Piezoresistive Property of Vanadium Dioxide Thin Film. Sens. Actuators A Phys..

[88] Kana Kana J.B., Vignaud G., Gibaud A., Maaza M. (2016). Thermally Driven Sign Switch of Static Dielectric Constant of VO_2_ Thin Film. Opt. Mater..

[89] Li W.W., Zhu J.J., Xu X.F., Jiang K., Hu Z.G., Zhu M., Chu J.H. (2011). Ultraviolet-Infrared Dielectric Functions and Electronic Band Structures of Monoclinic VO2 Nanocrystalline Film: Temperature-dependent Spectral Transmittance. J. Appl. Phys..

[90] Currie M., Mastro M.A., Wheeler V.D. (2017). Characterizing the Tunable Refractive Index of Vanadium Dioxide. Opt. Mater. Express.

[91] Wan C., Zhang Z., Woolf D., Hessel C.M., Rensberg J., Hensley J.M., Xiao Y., Shahsafi A., Salman J., Richter S. (2019). On the Optical Properties of Thin-Film Vanadium Dioxide from the Visible to the Far Infrared. Ann. Phys..

[92] Abdelkadir A.A., Victor J.L., Vignaud G., Marcel C., Sahal M., Maaza M., Chaker M., Gibaud A. (2023). Analysis of the Temperature Dependent Optical Properties of V1-xWxO_2_ Thin Films. Thin Solid Film..

[93] Sun X., Cai Q., Qi J., Liu B., Zheng Y., Zhang R., Li J., Wang S., Chen L., Lee Y. (2025). Temperature-Dependent Spectroscopic Ellipsometry and Modeling of the Optical Properties of Vanadium Dioxide Thin Films. Crystals.

[94] Morris B.S., Shayegan Z., Koch D., Palpant B., Chaker M. (2024). Developing an Analysis Procedure and Dispersion Model for Pristine and W-Doped VO_2_ Thin Films Using Density Functional Theory and Spectroscopic Ellipsometry. ACS Appl. Mater. Interfaces.

[95] Powell R.J., Spicer W.E. (1970). Optical Properties of NiO and CoO. Phys. Rev. B.

[96] Ukoba K.O., Eloka-Eboka A.C., Inambao F.L. (2018). Review of Nanostructured NiO Thin Film Deposition Using the Spray Pyrolysis Technique. Renew. Sustain. Energy Rev..

[97] Sawaby A., Selim M.S., Marzouk S.Y., Mostafa M.A., Hosny A. (2010). Structure, Optical and Electrochromic Properties of NiO Thin Films. Phys. B Condens. Matter.

[98] Sato H., Minami T., Takata S., Yamada T. (1993). Transparent Conducting P-Type NiO Thin Films Prepared by Magnetron Sputtering. Thin Solid Film..

[99] Kitao M., Yamada S., Hiruta Y., Suzuki N., Urabe K. (1988). Electrochromic Absorption Spectra Modulated by the Composition of WO_3_/MoO_3_ Mixed Films. Appl. Surf. Sci..

[100] Johansson M.B., Baldissera G., Valyukh I., Persson C., Arwin H., Niklasson G.A., Österlund L. (2013). Electronic and Optical Properties of Nanocrystalline WO_3_ Thin Films Studied by Optical Spectroscopy and Density Functional Calculations. J. Phys. Condens. Matter.

[101] Johansson M.B., Zietz B., Niklasson G.A., Österlund L. (2014). Optical Properties of Nanocrystalline WO_3_ and WO_3-x_ Thin Films Prepared by DC Magnetron Sputtering. J. Appl. Phys..

[102] Valyukh I., Green S., Arwin H., Niklasson G., Wäckelgård E., Granqvist C. (2010). Spectroscopic Ellipsometry Characterization of Electrochromic Tungsten Oxide and Nickel Oxide Thin Films Made by Sputter Deposition. Sol. Energy Mater. Sol. Cells.

[103] Kulikova D.P., Dobronosova A.A., Kornienko V.V., Nechepurenko I.A., Baburin A.S., Sergeev E.V., Lotkov E.S., Rodionov I.A., Baryshev A.V., Dorofeenko A.V. (2020). Optical Properties of Tungsten Trioxide, Palladium, and Platinum Thin Films for Functional Nanostructures Engineering. Opt. Express.

[104] Zhang B., Wang J., Jiang S., Yuan M., Chen X. (2024). Full Spectrum Electrochromic WO_3_ Mechanism and Optical Modulation via Ex Situ Spectroscopic Ellipsometry: Effect of Li+ Surface Permeation. Micromachines.

[105] Rasheed M., Barillé R. (2017). Optical Constants of DC Sputtering Derived ITO, TiO_2_ and TiO_2_:Nb Thin Films Characterized by Spectrophotometry and Spectroscopic Ellipsometry for Optoelectronic Devices. J. Non-Cryst. Solids.

[106] Wang X., Chen X., Zhang D., Chen J., Deng P., Zhong Z., Xiang Q., Li J., Li F., Liao Y. (2019). UV Radiation Cumulative Recording Based on Amorphous TiO_2_ Nanotubes. ACS Sens..

[107] Joost U., Šutka A., Oja M., Smits K., Döbelin N., Loot A., Järvekülg M., Hirsimäki M., Valden M., Nõmmiste E. (2018). Reversible Photodoping of TiO_2_ Nanoparticles for Photochromic Applications. Chem. Mater..

